# Antioxidant Activity and Neuroprotective Role of Docosahexaenoic Acid (DHA) Supplementation in Eye Diseases That Can Lead to Blindness: A Narrative Review

**DOI:** 10.3390/antiox10030386

**Published:** 2021-03-05

**Authors:** María Lafuente, María Elena Rodríguez González-Herrero, Stéphanie Romeo Villadóniga, Joan Carles Domingo

**Affiliations:** 1 Service of Ophthalmology, Hospital Universitario Virgen de la Arrixaca, El Palmar, E-30120 Murcia, Spain; mariaelenargh@gmail.com; 2 Service of Ophthalmology, Complejo Hospitalario Universitario de Ferrol, Ferrol, E-15405 A Coruña, Spain; stephimed@hotmail.com; 3 Department of Biochemistry and Molecular Biomedicine, Faculty of Biology, University of Barcelona, E-08028 Barcelona, Spain; jcdomingo@ub.edu

**Keywords:** omega-3 fatty acids, docosahexaenoic acid, glutathione, diabetic macular edema, glaucoma, oxidative stress, eye health

## Abstract

The objective of this narrative review is to provide updated evidence, based on data from experimental and clinical studies, of the prominent role of omega-3 polyunsaturated fatty acids (n-3 PUFAs) for a number of crucial mechanisms involved in counteracting cell damage induced by oxidative stress in eye diseases. This article is focused on the antioxidant and neuroprotective effects of docosahexaenoic acid (DHA), which have been assessed in different experimental models and clinical studies, particularly in proliferative diabetic retinopathy, age-related macular degeneration and glaucoma that are the most common eye diseases leading to severe vision loss. The mechanisms involved in the role of DHA in protecting human retinal pigment epithelial cells from oxidative stress as well as the interaction with glutathione (GSH) are also described. The review is intended to provide novel and salient findings supporting the rationale of the use of dietary supplementation with high-dose DHA (1050 mg/day) in the form of triglyceride as a potent antioxidant compound for improving the eye health. However, the overall clinical evidence for the use of dietary strategies based on supplementation with n-3 PUFAs in eye diseases linked to oxidative stress other than high-dose DHA triglyceride is both limited and inconsistent.

## 1. Introduction

Oxidative stress has been defined as a phenomenon resulting from an imbalance between production and accumulation of free radicals or reactive oxygen species (ROS) in cells and tissues and the ability of biological system to detoxify these reactive metabolites (i.e., protective mechanisms by antioxidants) [1]. ROS generated by biological systems as metabolic by-products include superoxide radicals, hydrogen peroxide, hydroxyl radicals, and singlet oxygen. Mitochondria under both pathological and physiological conditions are an important source of ROS. Superoxide radicals are produced by cellular respiration, by cyclooxygenases (COX) during the metabolism of arachidonic acid and by lipoxygenases (LOX) as well as by endothelial cells and inflammatory cells [2]. The oxidation products or nitrosylated products linked to ROS reduce biological activity with loss of energy metabolism, transport, cell signaling and a variety of detrimental effects on crucial cellular functions. Proteasome degradation targeted by these altered products contributes to further reduction of cellular function [3]. Although cell organelles have intrinsic ROS scavenging capacity, enzymatic components such as catalase (CAT), superoxide dismutase (SOD), glutathione peroxidase (GPx) and glutathione reductase (GR) are the mainly enzymatic components involved in the cell enzymatic antioxidant defensive system [4].

On the other hand, ROS are important signaling molecules involved in the progression of inflammation, with the production of oxidized proteins, glycated products and lipid peroxidation induced by oxidative stress resulting in an inflammatory response [5]. Key inflammatory events in which ROS are involved mainly include increased vascular permeability, leukocyte extravasation, phagocytosis and angiogenesis. However, ROS-induced effects on inflammation are multi-faceted and still remain unclear [6], but overproduction of ROS may result in cell and tissue injury and contributes to chronic inflammation as the underlying pathophysiological mechanism of a wide spectrum of neurodegenerative, cardiovascular and metabolic diseases.

## 2. Antioxidant Activity of Glutathione and Omega-3 Fatty Acids

Glutathione (GSH) is the most abundant low molecular weight thiol compound synthesized in cells and plays critical roles in protecting cells from oxidative damage and in maintaining redox homeostasis. GSH is also involved in other functions including apoptosis, detoxification and modulation of cell proliferation [7]. The first step in the synthesis of GSH is the production of γ-glutamylcysteine due to the combination of glutamate with cysteine. The second step to produce GSH includes the addition of glycine to the dipeptide with the action of the glutathione synthetase enzyme. The thiol (sulfhydryl) group of cysteine plays a role in GSH-related conjugation and reduction reactions, which are essential functions in the antioxidant activity of GSH. Although all cell types synthesize GSH, the liver remains the main GSH source in the body. Hepatocytes supply GSH found in plasma, which is used as a source of cysteine for GSH synthesis in other cells [7].

Glutathione is present in two forms: the disulfide-oxidized (glutathione disulfide, GSSG) form and the GSH thiol-reduced form. The redox-active thiol group modulates the antioxidant activity of GSH, becoming oxidized when target molecules are reduced by GSH. In response to different apoptotic stimuli, depletion of GSH is an early indicator of programmed cell death. Maintenance of the GSSG and harmful nitrogen and reactive oxygen species at very low levels together with GSH at a high level is attained by cellular redox homeostasis. The intracellular GSH:GSSG ratio is considered a marker of cellular toxicity. In a resting state and in normal conditions, the GSH:GSSG molar ratio exceeds 100:1, whereas it decreases to 10:1 (and even 1:1) in oxidative stress models [8].

**Figure 1:**
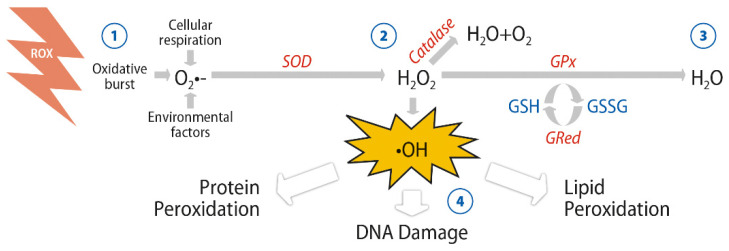
Anion superoxide (O_2_^●−^) is converted into hydrogen peroxide (H_2_O_2_) by superoxide dismutase (SOD) to avoid formation of the hydroxyl radical (^●^OH). Catalase and glutathione peroxidase (GPx) are converting the peroxide into molecular oxygen and water (H_2_O) by using electrons given by glutathione (GSH), thus avoiding oxidative harm onto the DNA, lipids, and proteins of the cell.

Cellular enzymatic mechanisms by which oxygenated free radicals are scavenged are shown in Figure 1.

While GSH is the most important antioxidant product synthesized in the cells, there are other antioxidant compounds that are obtained from the diet, such as vitamins E and C, selenium, carotenoids, lutein and zeaxanthin [9]. n-3 PUFAs can help to attenuate oxidative stress. The fish oil n-3PUFAs, eicosapentaenoic (EPA) acid and particularly, docosahexaenoic (DHA) acid, have demonstrated the most promising and consistent beneficial health effects, including anti-inflammatory, antiproliferative, antiangiogenic and antioxidant properties [10,11,12]. In common to other n-3 fatty acids, DHA can be synthesized from the plant-derived α-linoleic acid (ALA) found in components of the human diet, but the extent of conversion of ALA to DHA appears to be small. Seafood and products derived from seafood are the primary dietary sources of DHA, so that DHA intake is heavily influenced by fish consumption. Dietary supplementation with DHA is an alternative supply to endogenous synthesis of DHA to achieve optimum levels of n-3PUFAs in the body and maintain essential functions, mainly neuroprotective.

n-3 PUFAs have important physiological functions because of their presence as phospholipids in cell membranes, contributing to an optimum bilayer structure, which is necessary for intercellular communication and other highly differentiated functions [13]. n-3 PUFAs are primary precursors of bioactive lipid mediators, including eicosanoids, which have diverse paracrine and autocrine actions [10]. In nervous tissue, DHA is present in high concentrations, playing an essential role in neuroprotection and brain development [14,15]. In the retina, the highest concentration of DHA is found in the outer segments of rod photoreceptors where DHA plays a crucial role in retinal function [16] (see Section 3.1), which is in contrast to minor significance of EPA at ocular level.

### Enhanced Antioxidant Response of DHA and GSH

Experimental studies have provided some lines of evidence of an interrelationship in the antioxidant defense mechanisms exerted by DHA and GSH. In a model of human fibroblast culture, supplementation with 30 µmol/L DHA was accompanied by a large increase in intracellular GSH content in association with an elevated catalytic activity of glutathione reductase and glutathione-5-transferase [17]. Increased GSH contributed to reduce ROS as evaluated by a decreased accumulation of dicholorofluorescein inside cells. Apparently, this was the first report of a potent and specific effect of DHA for reducing oxidative stress in human fibroblasts [17].

In a rat pheochromocytoma cell line (PC12) culture, pretreatment with DHA (24 h) protected the cells from H_2_O_2_-induced oxidative damage by different mechanisms, including the regulation of the nuclear factor erythroid 2 like 2 (NFE2L2) and its downstream target protein, heme-oxygenase-1 (HO-1), and an increase in intracellular levels of enzymatic antioxidants such as SOD and GPx [18]. Interestingly, it has been recently shown that n-3 PUFAs may suppress pro-oxidant activity by upregulating genes encoding cytoprotective antioxidant proteins such as heme oxygenase 1 (HO-1) and glutathione peroxidase (GPx) [19]. In a study of dopaminergic SM4741 cells, the toxic effect of paraquat related to an increase of intracellular ROS content in different organelles was ameliorated by pretreatment with DHA, which also increased glutathione reductase and the accumulation of the GSH pool by enhancing GSH homeostasis regulated by NFE2L2 protein levels [20].

**Figure 2:**
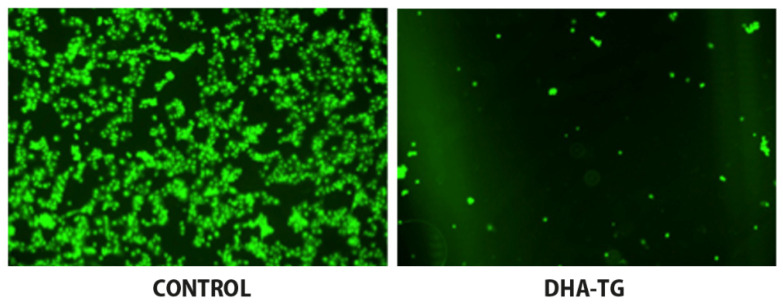
Retinal ARPE-19 cells (×10 magnification) showing disappearance of oxidation of fluorescent probes by DHA triglyceride (DHA-TG) (right) as compared with control (left) as indicative of removal of intracellular ROS (reactive oxygen species).

DHA has been shown to be involved in maintaining healthy retinal cells. In an experimental model of retinal pigment epithelial cells (ARPE-19 cells) under oxidative stress by exposure to H_2_O_2_, pretreatment with DHA augmented GSH levels regardless of H_2_O_2_ levels and increased ARPE-19 cell viability; viability was also enhanced by pretreatment with lutein and xanthine [21]. Additionally, treatment with DHA enhanced n-3 PUFAs enzymatic oxidation and these effects were not influenced by the carotenoids [21]. In another model of ARPE-19 cells, cellular damage associated with lipid peroxidation and DNA breaks was induced with a free radical initiator (2,2′-azobis-(2-amidinopropane)-dihydrochloride, AAPH) and buthionine sulfoximine (BSO). BSO is a sulfoximine derivative which reduces the levels of GSH. The effects of a concentrated DHA in the form of triglyceride (DHA-TG), having a high antioxidant activity patented to prevent cellular oxidation damage [22]) was assessed by measurement of intracellular pool of GSH in AAPH- and BSO-treated cells. It was shown that nearly 50% of ROS could be removed when cultures were simultaneously incubated with DHA-TG, which promoted the endogenous antioxidant defense through an increase of the intracellular GSH (Figure 2) [23]. This study provides evidence of the potent effect of DHA-TG for decreasing oxidative stress involving GSH on pigment epithelial cells of the human retina.

## 3. Protective Effects of DHA against Oxidative Stress in Eye Structures

### 3.1. Retinal Cells and Photoreceptors

In the human body, the retina has the highest oxygen consumption per gram of tissue, requiring large amounts of adenosine triphosphate (ATP) to support cellular functions. This high metabolism makes the retina especially vulnerable to oxidative stress damage. Numerous studies have shown that ROS contributes to vascular endothelial dysfunction and retinal neural degeneration [24]. Moreover, inflammation in the retina and neuron degeneration has been linked to excessive ROS formation, which can directly modify cellular molecules and impair their function. The production of inflammatory cytokines, such as interleukin-6 (IL-6), IL-1β and tumor necrosis factor-alpha (TNFα), as well as the activation of transcription factors, such as nuclear factor (NF)-kappa B (NF-κB) can be stimulated by ROS causing inflammation and cell death [25]. Retinal endothelial dysfunction caused by accumulation of ROS impairs the balance of nitric oxide (NO) metabolism and the responsiveness of smooth muscle cells and vascular endothelial cells to physiological stimuli. Reduced endothelium-dependent vasodilation and a prothrombotic and proinflammatory state are characteristic features of endothelial dysfunction [25].

**Figure 3:**
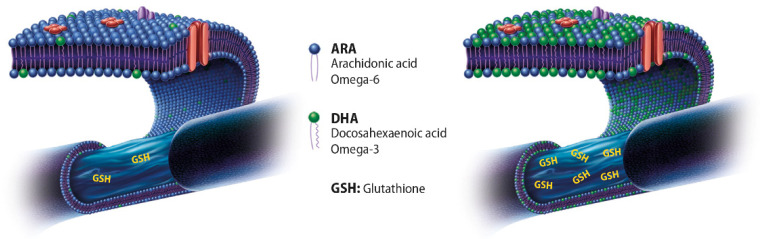
Photoreceptors, as well as other neuron-like cells, have an intensified metabolism, thus cumulating larger amounts of reactive oxygen species (ROS) in their cytoplasm. Moreover, DHA and arachidonic acid (AA) together are almost one fifth of the dry weight. GSH is the main antioxidant produced in the cell’s cytoplasm to scavenge the ROS to avoid membrane oxidation. Appropriate amounts of DHA in the cell membrane are upregulating GSH production, and this is to prevent the oxidation of the double bonds present in the membrane phospholipids. The decrease of photoreceptor membrane DHA after a currently Western diet may result in impaired cellular antioxidant function that can be corrected by increased intake of n-3 PUFAs such as DHA-TG.

Lipids are structural components of cell membranes, provide energy storage, and act as signaling molecules. n-3 PUFAs are essential for neural development of the brain and different structures, including the retina. n-3 PUFAs cannot be efficiently synthesized by the human body, so that adequate amounts depend on the consumption of foods rich in these compounds, such as fish and fish oil products (Figure 3). DHA accounts for approximately 20% of the retinal weight and affects the development and survival of neurons and retinal vascular cells [24]. DHA is the dominant fatty acid of retinal phospholipids and plays a significant role in maintaining retinal integrity [25], also related to the increased production of GSH (Figure 3).

Cell membranes of the rod outer segment of retinal photoreceptors have a high content of DHA (50–70% of fatty acids) since DHA is a component of phospholipids that cluster around rhodopsin, which is the protein that receives the light signal. When the light signal is received, a phototransduction cascade is initiated through activation of a conformational change of rhodopsin, which is facilitated by the presence of DHA within the membrane [26]. On the other hand, the retina has an abundance of DHA-containing phospholipids (DHA-PL). In a model of lysophosphatidic acid acyltransferase 3 (LPAAT3)-knock-out mice, abnormalities in the retinal layers following loss of DHA-PL were observed, such as incomplete elongation of the outer segment and decreased thickness of the outer nuclear layers, as well as disordered disc shape in photoreceptor cells [27].

In an in vitro study of ARPE-19 cells, the ability of n-3 PUFAs to rescue retinal pigment epithelial cells from the oxidative and inflammatory conditions seen in diabetic retinopathy (DR) was evaluated [28]. It was found that EPA and DHA, especially in the form of triglyceride, produced favorable effects on retinal cells by increasing cell viability and proliferation, reducing the production of ROS, and decreasing oxidative damage caused by H_2_O_2_ [28]. In a study of pure rat retinal neurons in culture subjected to oxidative stress with paraquat and H_2_O_2_, direct neuroprotection of photoreceptors from apoptosis by xanthophylls and DHA was found, with increased expression of opsin (light-sensitive protein constituent of rhodopsin) suggesting enhancement of photoreceptor differentiation [29]. In this model, it has been shown that DHA activates intracellular mechanisms that prevent loss of mitochondrial membrane integrity, which is an essential step in the apoptotic death of these cells [30]. In another study of cultured rat ganglion cell line (RGC-5 cells), DHA showed concentration-dependent radical scavenging activity for H_2_O_2_, superoxide anion, and hydroxyl radical, and inhibition of the decrease in cell viability induced by H_2_O_2_ [31]. It should be noted that RGC-5 cells were indeed of mouse origin and have been characterized as the cell line 661W, a mouse SV-40 T antigen transformed photoreceptor cell line [32]. On the other hand, DHA enhanced the synthesis of 10,17S-docosatriene (neuroprotectin D1 [NPD1]), which protected retinal pigment epithelial cells from oxidative stress-induced apoptosis [33] via PI3K/Akt and mTOR/p70S6K pathways [34].

Finally, in an in-depth review of the role of DHA in the retina by SanGiovanni and Chew [35], it has been shown that photoreceptor membrane function can be affected by biophysical and biochemical properties of DHA by altering lipid phase properties, permeability, thickness and fluidity. The mechanisms of retinal cell signaling involved in phototransduction can be also affected by tissue DHA status. DHA may also be involved in signaling cascades related to rhodopsin function and enhanced activation of membrane-bound retinal proteins. In addition, DHA has shown the pleiotropic actions, including the inhibitory effect on the activation of NF-κB with reduction of associated cytokines (IL-6, IL-1β, TNFα, vascular endothelial growth factor [VEGF]), and stimulation of inflammation resolving docosanoids (resolvins and protectins) [10].

All these findings support dietary supplementation with DHA to ameliorate the effect of oxidative stress damage in eye diseases that can severely compromise vision, such as DR, age-related macular degeneration and glaucoma, in which oxidative stress has a pathogenetic role among other inflammatory, proliferative, and proangiogenic contributing pathways.

### 3.2. Trabecular Meshwork and Intraocular Pressure

The trabecular meshwork is a specialized eye tissue that regulates the outflow of the aqueous humor and controls intraocular pressure (IOP). Cells of this biological filter are crucial for maintaining homeostasis of the whole outflow system through which the aqueous humor reaches the Schlemm canal. There is increasing evidence of the pathogenetic role of oxidative DNA damage affecting the trabecular meshwork cells in the development of glaucomatous optic neuropathy [36,37,38,39,40], and its relation with glutathione S-transferase genetic polymorphisms and lack of the *GSTM1* gene, which catalyzes neutralization of free radicals by conjugation with GSH [40,41].

Reduced ocular blood flow associated with red blood cell membrane abnormalities have been implicated in the pathogenesis of primary open-angle glaucoma (POAG). In a study of lipid composition of red blood cell membranes in patients with POAG, reduced erythrocyte levels of phosphatidyl-choline (PC) carrying docosahexaenoic acid (DHA) was reported [42,43]. In Sprague-Dawley rat dams, induced IOP elevation associated with a deficient n-3 PUFAs diet caused dysfunction of retinal ganglion cell activity, and the combination of these factors (dietary manipulation and IOP stress) showed a cumulative effect [44]. The authors concluded that sufficient dietary n-3 PUFAs improves retinal ganglion cell function making it less susceptible to IOP insult, which may have implications for glaucoma [44]. In a DBA/2J mouse model of hereditary glaucoma, supplementation of n-3 PUFAs had neuroprotective effect in the retinas, with downregulation of TNFα and IL-18 [45].

Different clinical studies have investigated the implications of oxidative stress in patients with POAG. In a study of 40 patients with POAG and 60 healthy controls, significantly higher levels of plasma malondialdehyde (MDA) as a marker of oxidative stress were found in patients with POAG [46]. In a sample of 21 patients with POAG and 34 age- and gender-matched control subjects, patients with glaucoma showed significantly lower levels of GSH suggesting a general impairment of the antioxidative defense [47]. In another study of 206 patients with POAG and 126 controls, lower systemic antioxidant capacity in glaucoma patients was demonstrated as measured by plasma levels of reactive oxygen metabolites, antioxidant capacity, and thiol-antioxidant capacity [48]. In a study in which DNA damage markers and the antioxidant status of serum and aqueous humor were measured from 28 patients with glaucoma and 27 patients with cataracts at the time of surgery, aqueous humor and serum levels 8-hydroxy-2′-deoxyguanosine (8-OHdG), an established marker for oxidative stress-induced DNA damage were significantly higher in glaucoma patients, whereas aqueous and serum total antioxidant status was significantly lower [49]. A further study that evaluated DNA damage in terms of 8-OHdG in specimens of trabecular meshwork from 42 patients with glaucoma and 42 controls of similar age and sex, also showed significantly higher levels of 8-OHdG in glaucoma patients [50]. Moreover, the *GSTM1* genotype assessed by polymerase chain reaction in DNA samples showed that the *GSTM1*-null genotype was significantly more common in patients with glaucoma, who also showed significantly higher levels of 8-OHdG as compared with patients with *GSTM1*-positive genotype [41].

Taken together, these studies suggest that dietary supplementation with DHA could modulate impaired antioxidant status associated with glaucomatous damage.

## 4. Eye Benefits of Dietary Supplementation with DHA

### 4.1. Diabetic Retinopathy and Macular Edema

Severe vision loss in DR is seen in diabetic macular edema and proliferative DR, which is characterized by retinal neovascularization. The crucial factor leading to vision loss and eventually blindness is chronic hyperglycemia due to poorly controlled blood glucose levels. Hyperglycemia leads to the activation of metabolic pathways that involve inflammation, oxidative damage, and neurovascular dysfunction. The duration of diabetes and poor glycemic control are key factors increasing the prevalence of DR. Approximately, 33% of diabetic patients present signs of DR and 10% have vision-threatening stages of DR [24].

Oxidative stress forms part of the multifactorial pathogenesis of DR and diabetic macular edema [51,52] and provides the rationale of dietary supplementation with DHA and other naturally occurring antioxidants, such as carotenoids and xantophylls. However, DHA exhibits a vision-regulating role ensuring proper functioning of rhodopsin pigment present in the retinal photoreceptor cells. DHA is also a strong anti-inflammatory agent and inhibits the expression of VEGF, decreasing the production of superoxide radicals involved in the VEGF signaling pathway [51]

Of the three n-3 PUFAs, ALA, EPA and DHA, which can be used for delaying/inhibiting the various underlying mechanisms induced during hyperglycemia focusing on DR, DHA exhibits the widest range of anti-inflammatory mediators, besides decreasing the formation of free radicals and inducing expression of endogenous antioxidant enzymes and remarkably preventing retinal angiogenesis by downregulation of the expression of angiogenic agents especially VEGF [51].

Real-life outcomes of dietary supplementation with high-dose DHA-TG in DR were assessed in a prospective controlled study of 12 asymptomatic patients with nonproliferative DR (*n* = 24 eyes) and 12 healthy controls (*n* = 24 eyes) [53]. Variables measured were macular sensitivity and integrity, visual acuity, macular thickness by optical coherence tomography (OCT), plasma total antioxidant capacity (TAC), and DHA content in the erythrocyte membrane. Participants in the experimental group received a high-rich DHA-TG (1050 mg/day) supplementation, whereas controls received no treatment but were blinded regarding the existence of an experimental group. Of note is that TAC in plasma was measured using a commercially available kit, with uric acid equivalent used to calculate cooper-reducing equivalent values (µM Cu-reducing equivalent). High TAC values reflect high antioxidant capacity, i.e., greater protection. The duration of treatment was 90 days. Salient results included statistically significant differences in macular function in favor of the experimental group, with plasma TAC values and DHA content of the erythrocyte membrane increasing significantly in the experimental group only. In conclusion, supplementation with high-dose DHA-TG at an early stage of DR improved macular function measured by microperimetry and increased the antioxidant status capacity.

Plasma TAC was also determined in a 3-year randomized single-blind controlled trial of intravitreal injection of ranibizumab combined with oral supplementation with DHA-TG in diabetic patients with macular edema [54,55]. Briefly, all patients had type 2 diabetes with decreased vision due to diabetic macular edema (DME) (1 mm thickness in the central subfield OCT). In this trial, patients were randomized to treatment with ranibizumab (loading dose 0.5 mg/0.05 m for the first 4 months followed on as as-needed treatment) alone (control group) or combined with high-rich DHA-TG supplementation (1050 mg/day) (DHA group). The dietary supplement also contained 127 mg of EPA. TAC was expressed as µM Cu-reducing equivalent. At 2 years with 33 patients and 42 eyes analyzed in the control group, and 34 eyes and 29 patients in the DHA group, the decrease in central subfield macular thickness was statistically significant in favor of the DHA supplementation group, which was also observed at 3 years. Visual outcomes, however, were similar in both two study groups, either at 2- or 3-year assessments. As expected, differences in plasma TAC and erythrocyte membrane DHA content were all significant in favor of the DHA supplementation group. This study shows the beneficial effect of DHA supplementation in terms of anatomical improvement in DME.

Findings of a substudy from the PREDIMED randomized clinical study (Prevention with Mediterranean Diet) showed that in older and middle-age subjects with type 2 diabetes, intake of at least 500 mg/day of dietary n-3 PUFAs (minimum two weekly servings of oily fish) was associated with a 46% risk reduction of incident sight-threatening DR as compared with patients not fulfilling this dietary recommendation [56]. In this study, however, dietary intake of n-3 PUFAs was estimated using a validated dietary questionnaire in which the consumption of 8 different types of seafoods were included, but information regarding the consumption of specific amounts of EPA and DHA is not provided. Additionally, differences in the risk of DR could be related to other lifestyle factors, as well as to the fact that participants who met n-3 PUFAs consumption target tended to be younger and showed a lower prevalence of hypertension (or antihypertensive treatment) and used less insulin than participants not fulfilling the dietary target recommendation.

### 4.2. Age-Related Macular Degeneration

Similar studies of dietary supplementation with high-dose DHA-TG in age-related macular degeneration (AMD) have not been carried out, but results reported from a variety of research are inconsistent regarding the effects on omega-3 fatty acids on visual acuity, progression and development of drusen or the presence of geographic atrophy [57]. However, it has been reported that high erythrocyte content of EPA/DHA is a protective factor against AMD compared with permanently low EPA/DHA levels [58]. Results of the phase 3 randomized controlled AREDS2 study in which there was no overall additional benefit from adding n-3 PUFAS or lutein and zeaxanthin to the original AREDS formulation (vitamins C and E, beta-carotene, zinc oxide, cupric oxide) in reducing the risk of advanced AMD [59] merits a comment. In contrast to the use of a highly concentrated dose of DHA-TG (1050 mg/day) in the randomized studies of diabetic macular edema [54,55], participants in the AREDS2 study received 10 mg lutein, 20 mg zeaxanthin, and 650 mg of EPA and 350 mg of DHA in the form of ethyl esters. It should be noted that the daily dose of DHA in AREDS2 study was markedly inferior to that used in the randomized trials of diabetic macular edema [54,55], and that the ethyl ester formulations of n-3 PUFAS created in the process of transesterification have shown lower bioavailability than chemical triglyceride binding [60].

It is interesting to discuss the effect of DHA administered in combination with lutein/zeaxanthin. An experimental study in quails showed that supplementation with lutein/zeaxanthin prevented light-induced photoreceptor cell death [61]. In a clinical study of 100 healthy participants (200 eyes) aged 40–70 years randomized in a 1:1 ratio to receive daily lutein or lutein/DHA for 3 months, macular pigment optical density (MPOD) showed significantly higher values in the lutein/DHA group than in the lutein alone group, as well as higher lutein levels in plasma, so that this study highlights the relevance of the adjunctive role of DHA for a better lutein bioavailability [62]. In a 4-month study of 49 women (aged 60–80 years) randomized to lutein (12 mg/day), DHA (800 mg/day), lutein + DHA, or placebo, lutein alone increased MPOD eccentrically, DHA supplementation alone resulted in central increases in MPOD, and the combination of supplements had a combined effect on the MPOD spatial profile. In addition, DHA facilitated accumulation of lutein in the blood and macula [63].

The Veterans LAST (Lutein Antioxidant Supplementation Trial) was a prospective, 12-month, randomized, double-masked, placebo-controlled trial aimed to assess whether nutritional supplementation with lutein or lutein together with antioxidants, vitamins, and minerals improves visual function and symptoms in atrophic AMD [64]. There were three study groups: patients in group 1 received lutein 10 mg (L); in Group 2, a lutein 10 mg/antioxidants/vitamins and minerals broad spectrum supplementation formula (L/A) (n-3 PUFAs were not included); and in Group 3, a maltodextrin placebo (P) over 12 months. In patients treated with lutein alone or lutein together with other nutrients, there was a significant increase in eye macular pigment optical density. Visual acuity and contrast sensitivity also improved. In a further analysis of the same study population, it was observed that patients with lower baseline values of macular pigment optical density were those most likely to benefit from either the lutein or the lutein plus antioxidant supplementation [65]. Accordingly, it may be inferred that if a deficiency in macular pigment optical density is accurately diagnosed, effective interventions should be able to re-establish this prophylactic barrier.

Other studies of n-3 PUFA supplementation in AMD besides AREDS and AREDS2 have been carried out. In a large study of 263 patients over 55 years of age with early lesions of age-related maculopathy and randomized to receive either 840 mg/day DHA and 270 mg/day EPA from fish oil capsules or the placebo (olive oil capsules) for 3 years, no differences were observed in the occurrence of choroidal neovascularization in the study eye [66]. In a prospective study over more than 2 decades of follow-up of 75,889 women from the Nurses’ Health Study and 38,961 men from the Health Professionals Follow-up Study, the intake of EPA + DHA ≥ 350 mg/day (697 mg/day for the fifth quintile) or fatty fish ≥ 2 servings/week showed a moderate reduction in the risk of visually significant AMD, but was inconclusive regarding protection against development of advancement AMD [67].

### 4.3. Glaucoma

Antioxidant supplementation in patients with glaucoma has been associated with beneficial effects as extensively reported in a recent comprehensive review of 19 clinical trials. Data, however, are difficult to interpret because of differences among studies of types of glaucoma patients, study outcomes, and antioxidant substrates [68].

There is limited information on the use of high-dose DHA supplementation in glaucoma. A prospective randomized open-label controlled study in patients with pseudoexfoliative glaucoma (PEX) was conducted to assess improvement of antioxidant protection associated with high-rich dietary supplementation with DHA triglyceride [69]. Patients were assigned to active supplementation (DHA-TG 1050 mg/day) (*n* = 23) or to a control group (*n* = 24) without dietary intervention. The duration of treatment was 6 months. Biochemical analyses in this trial included plasma TAC (µM Cu-reducing equivalent values), plasma MDA, and erythrocyte membrane content of DHA (% total fatty acids). In the DHA group, mean IOP in both eyes decreased significantly at the end of treatment. In addition, as compared with baseline TAC levels and DHA content in the erythrocyte membrane increased significantly, whereas MDA decreased significantly in the DHA group only. Although the duration of treatment was limited to 6 months, decreases in IOP are suggestive of the clinical value of reducing oxidative stress with a high-rich DHA supplement in patients with PEX. In other studies, oral supplementation with high-dose DHA triglyceride achieved a reduction in oxidative stress markers and a significant improvement in symptoms and signs of eye dryness present in glaucoma patients [70,71].

## 5. Conclusions

Eyes are by far the most important organs of sense, and loss of vision in particular affecting the central fovea, has a profound impact on all aspects of an individual’s life. The identification of underlying cellular and molecular pathogenetic mechanisms in eye diseases has been a crucial advancement in the development of effective treatment strategies for preventing potential causes of blindness. The involvement of local oxidative stress and oxidative DNA damage in the underlying mechanisms of retinal diseases or glaucoma that can lead to blindness supports the rationale of the use of antioxidants in the management of these conditions. Randomized controlled studies of dietary supplementation with high-dose DHA in the form of triglyceride have provided evidence of the significant beneficial effects of n-3 PUFAs in the care of patients with DR, macular edema, and optic neuropathy associated with glaucoma. However, the overall clinical evidence for the use of dietary strategies based on supplementation with PUFAs in eye diseases linked to oxidative stress other than high-dose DHA triglyceride is both limited and inconsistent.

## Abbreviations

AA	arachidonic acid
AAPH	2,2′-azobis-(2-amidinopropane)-dihydrochloride
ALA	α-linoleic acid
AMD	age-related macular degeneration
ATP	adenosine triphosphate
BSO	buthionine sulfoximine
CAT	catalase
COX	cyclooxygenases
DHA	docosahexaenoic acid
DHA-PL	DHA containing phospholipids
DHA-TG	DHA triglyceride
DME	diabetic macular edema
DR	diabetic retinopathy
EPA	eicosapentaenoic acid
GPx	glutathione peroxidase
GR	glutathione reductase
GSH	glutathione
GSSG	glutathione disulfide
H_2_O	water
H_2_O_2_	hydrogen peroxide
IL	interleukin
IOP	intraocular pressure
LOX	lipoxygenases
LPAAT3	lysophosphatidic acid acyltransferase 3
MDA	malondialdehyde
MPOD	macular pigment optical density
n-3 PUFAs	omega-3 polyunsaturated fatty acids
NFE2L2	nuclear factor erythroid 2 like 2
NF-κB	nuclear factor (NF)-kappa B
NO	nitric oxide
NPD1	neuroprotectin D1
OCT	optical coherence tomography
8-OHdG	8-hydroxy-2′-deoxyguanosine
PC	phosphatidyl-choline
PEX	pseudoexfoliative glaucoma
POAG	primary open-angle glaucoma
ROS	reactive oxygen species
SOD	superoxide dismutase
TAC	total antioxidant capacity
TNFα	tumor necrosis factor-alpha
VEGF	vascular endothelial growth factor
